# Macromolecular crystallography from an industrial perspective – the impact of synchrotron radiation on structure-based drug discovery

**DOI:** 10.1107/S1600577524012281

**Published:** 2025-02-06

**Authors:** H. Käck, T. Sjögren

**Affiliations:** a Protein Sciences, Structure and Biophysics, Discovery Sciences, BioPharmaceuticals R&D, AstraZeneca, Pepparedsleden 1, SE-431 50Gothenburg, Sweden; University of Manchester, United Kingdom

**Keywords:** structure-based drug design, drug discovery, synchrotron data collection, protein structures

## Abstract

Structure-based drug design has been an integral part of drug discovery for over three decades, contributing to the development of numerous approved drugs. Here we discuss the evolution, as well as the current state, of structure-based drug design within the pharmaceutical industry, using data from AstraZeneca’s internal repository for crystal structures to provide additional context. Over the past 20 years, the company has transitioned from a mixed in-house and synchrotron data collection model to a ‘synchrotron-only’ approach, enabled by technological advancements at synchrotron facilities. We provide real-world examples of structure delivery to projects, including a high-throughput project and a case where a single structure was pivotal for discovering a candidate drug. We conclude that, despite recent developments in single-particle cryo-EM and deep-learning structure prediction methods, macromolecular crystallography remains a critical tool for drug discovery.

## Abstract 2

Structure-based drug design has been a critical component of drug discovery for over 30 years, contributing to numerous approved drugs. The paper discusses the approach taken by AstraZeneca (Sweden) to synchrotron data collection for supporting a large portfolio of drug discovery projects and provides examples to demonstrate the continued importance of experimental structures.

## 1. Introduction

Small-molecule drug discovery relies on identifying compounds interacting with a target protein in a way which leads to a favourable biological effect while being safe. Crystal structures detailing how an inhibitor binds to an enzyme or how a modulator interacts with its receptor are powerful tools in this context since they enable rational design of novel and better molecules. As such, structural information has the ability to inform and accelerate drug discovery. An early impactful example of structure-based design was the development of HIV protease inhibitors in the 1990s (Blundell & Pearl, 1989 ▸; Dorsey *et al.*, 1994 ▸). Development of classical computational chemistry tools for drug design goes hand in hand with access to high-quality structures and have since only augmented the value of structural information (Batool *et al.*, 2019 ▸; Wu *et al.*, 2023 ▸). Structure-based drug design has therefore been an integral part of the drug discovery toolbox for over three decades (van Montfort & Workman, 2017 ▸) and has contributed to the development of numerous approved drugs (Verma & Prabhakar, 2015 ▸). A recent analysis shows that 80% of drugs for anti-cancer treatment approved in the period 2019–2023 were designed with structural information at hand (Burley *et al.*, 2024 ▸). An example is Capivasertib, an AKT inhibitor that was recently approved for treatment of breast cancer, which was discovered by fragment-based drug discovery and structure-based design (Addie *et al.*, 2013 ▸; Saxty *et al.*, 2007 ▸; Caldwell *et al.*, 2008 ▸).

The concept of high-throughput crystallography and streamlining the crystallographic workflow has been around since the early 2000s. Method development was largely driven by structural genomics initiatives (Grabowski *et al.*, 2016 ▸; Joachimiak, 2009 ▸) and the advent of fragment screening by crystallography (Blundell *et al.*, 2002 ▸). In addition, technical improvements at synchrotron facilities have pushed the boundaries for high-throughput crystallography and broadened its applicability from a few favourable cases to a wide range of drug targets. Developments such as stable beams, fast detectors, effective sample changers with ever improving speed and reliability in combination with automated crystal characterization and data collection, and importantly, the opportunity to collect data remotely, have continually changed the way in which we have been collecting X-ray diffraction data over the past two decades (Helliwell & Mitchell, 2015 ▸; Keefe & Stoll, 2019 ▸; Grabowski *et al.*, 2021 ▸).

AstraZeneca has maintained a strong focus on structural biology since the mid-1990s. Today, the company has dedicated crystallography teams that collectively deliver around 800 unique protein–ligand complex structures per year, in addition to a group focused on cryo-EM.

While the crystallography team in Cambridge primarily supports the discovery of oncology therapeutics, the Gothenburg-based team focuses on a range of non-oncology therapeutic areas, including respiratory and immunology, cardiovascular, renal, metabolism, and neuroscience.

By interrogating the in-house structure repository, we can analyse the delivery of crystal structures to projects over time. This allows us to discuss the utility of macromolecular crystallography across the drug discovery value chain and describe workflows that enable sufficient capacity and timely delivery of structural information to maximize project impact. Furthermore, we substantiate these insights by providing relevant project examples.

## 2. 20 years of synchrotron data collection

The crystallography team at the AstraZeneca Gothenburg site (AstraZeneca Gothenburg) has maintained a repository of X-ray data collection records since the early 2000s. Over the 20-year period from 2004 to 2023, this repository contains 3717 unique structures that were delivered across 186 different non-oncology therapy area projects. Additionally, metadata are available for 22972 X-ray datasets collected at synchrotrons, including 20079 datasets that did not result in a structure.

**Figure 1:**
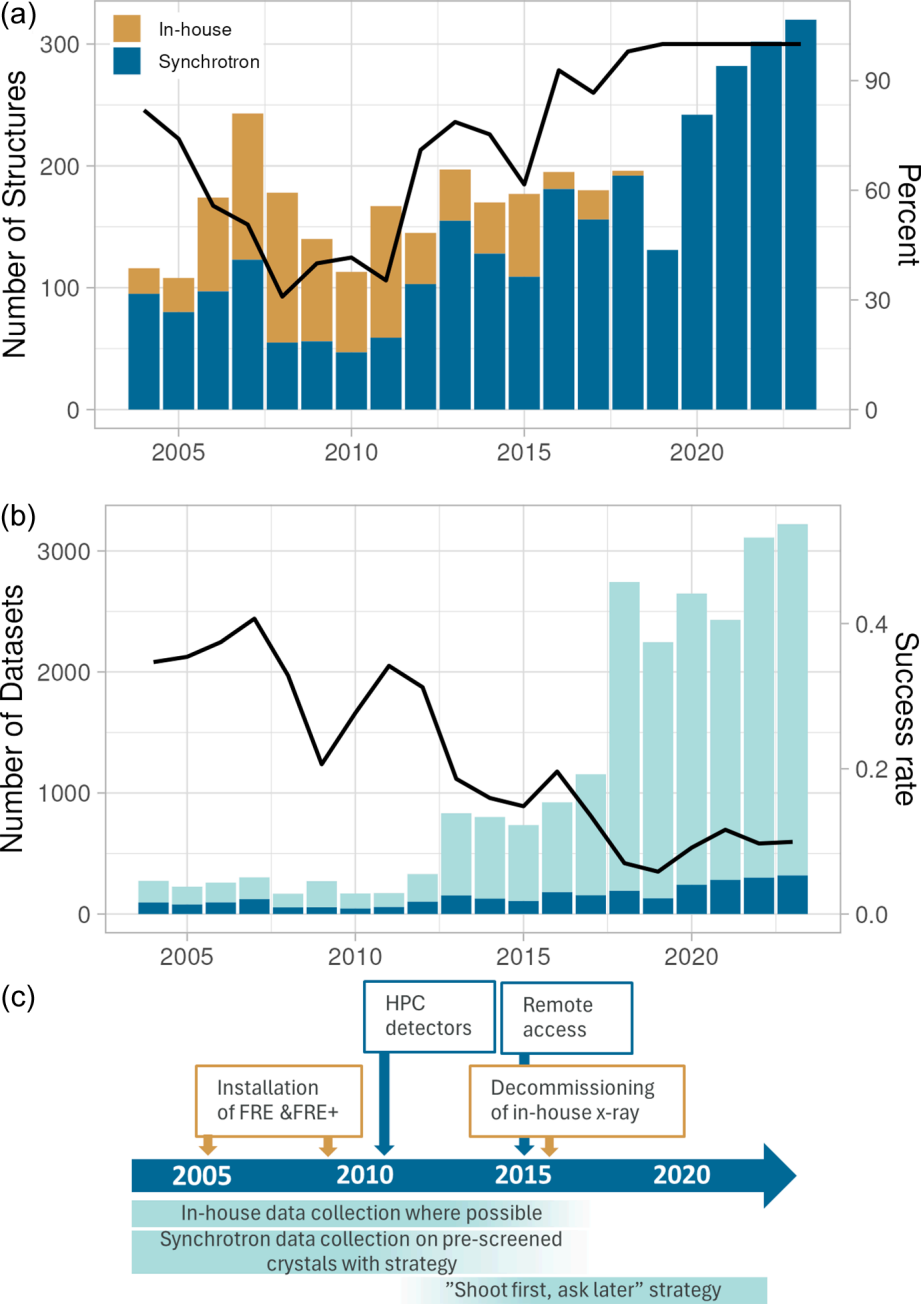
Structure determination and data collection statistics for 2004–2023. (*a*) Number of unique structures delivered per year, coloured according to whether data were collected on an in-house rotating anode or at a synchrotron. The fraction of structures determined from synchrotron data are shown as a solid line. (*b*) Number of datasets collected at synchrotrons per year. Datasets resulting in a unique structure are shown in dark blue. The success rate (number of structures per total number of datasets) is shown as a solid line. (*c*) Key events impacting the data collection strategy and choice of source.

The number of unique structures delivered between 2004 and 2023 has shown significant year-to-year fluctuations [Fig. 1 ▸(*a*)]. However, the three-year moving average has increased by more than 100%. When examining the number of synchrotron-collected datasets, we observe a more than tenfold increase over the same period [Fig. 1 ▸(*b*)]. Conversely, the fraction of datasets resulting in a structure (success rate) has decreased from over 35% to around 10%. The observed trends in both the number of structures and the datasets reflect changes in our ways of working, driven by technological advancements as well as strategic decisions [Fig. 1 ▸(*c*)].

In the early 2000s the throughput at synchrotrons was limited, where the total time for collecting a dataset, including sample mounting and alignment, varied between 20 min and several hours depending on the sample and beamline. We therefore screened every crystal to be shipped for synchrotron data collection on our in-house rotating anode (R-axis, Rigaku). At the synchrotron, the collection strategy was carefully planned based on a low number of test images. With 10–15 synchrotron shifts per year, the turnaround time for structures relying on synchrotron data was typically unfavourable, and therefore the ability to collect data on an in-house X-ray source was preferred whenever possible. The installation of an FRE rotating anode (Rigaku) in 2005, followed by an FRE+ (Rigaku) in 2009, enhanced our ability to collect data in-house to sufficient resolution resulting in a temporary shift towards more in-house-derived structures [Fig. 1 ▸(*a*)]. From our perspective, one of the most important developments at the synchrotrons was the introduction of hybrid photon counting (HPC) detectors. This shift is clearly visible in our data as a steep increase in number of images per dataset as the HPC detectors allowed for fine slicing of data (Fig. S1 of the supporting information). The mean recorded resolution also goes down at this time (not shown); however, factors like how data were cut may make this observation ambiguous. The HPC detectors, in combination with sample changers, massively increased the speed of data collection, such that a full data collection could be completed within minutes, often before diffraction can be visualized to users (Förster *et al.*, 2019 ▸). This led to a ‘shoot-first-ask-questions-later’ strategy, where 180° of data were collected for all crystals (except those known to have a *P*1 symmetry). The increased throughput contributed to a strategic decision to decommission our in-house X-ray sources in 2016. Since then, we rely on a ‘synchrotron only’ model for X-ray data collection. Decommissioning the in-house X-ray equipment, eliminating the possibility of pre-screening crystals, in combination with the ‘shoot-first-ask-questions-later’ strategy resulted in a steep decrease in data collection success rate [Fig. 1 ▸(*b*)]. The ‘unsuccessful’ datasets in the early 2000s reflect only structures with no ligand bound and a low number of redundant datasets, whereas today we routinely collect datasets for multiple crystals from the same protein–ligand complex, except for a few very favourable cases. We also do not stop, or refrain from collecting a full dataset due to poor diffraction, because the feedback on data quality is typically slower than collecting the data.

**Figure 2:**
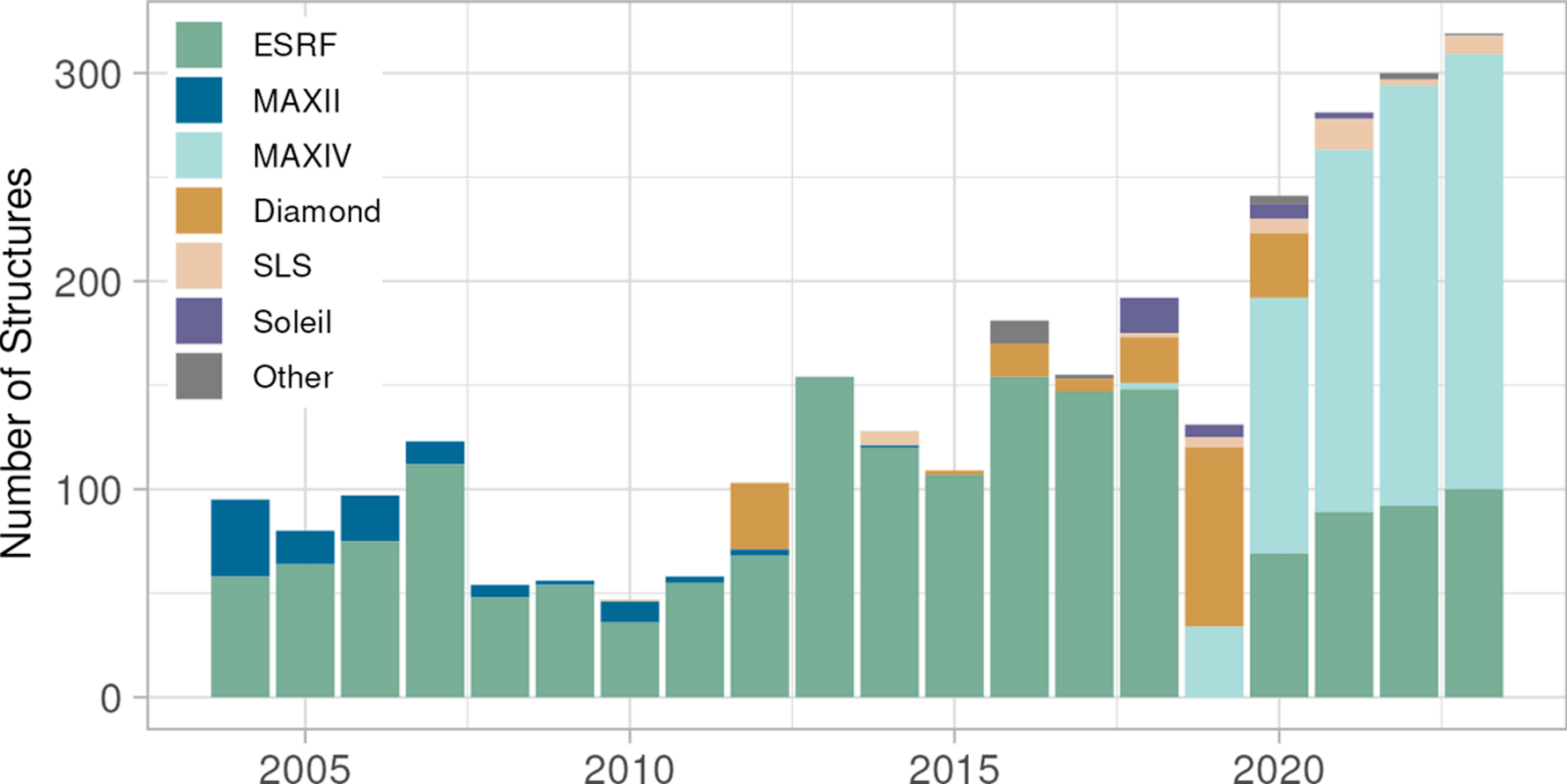
Number of structures derived from synchrotron data in the period 2004–2023. Structures are coloured according to the synchrotron. Historically, ESRF has been our main synchrotron of choice, but MAXIV has gained popularity in recent years mainly due to reliable shipping.

Due to our reliance on synchrotrons, we use multiple facilities to be able to collect data all year around on a near weekly basis, and to minimize our exposure to risks of technical failures (Fig. 2 ▸). We mainly used MAX IV, its predecessor MAX II and ESRF. Soleil and SLS are used for covering periods where both ESRF and MAX IV are unavailable for users. Diamond Light Source was used during the refurbishment of ESRF and before MAX IV came online. With all synchrotrons offering fit-for-purpose beamlines for X-ray crystallography, the choice of synchrotron is mainly based on practical considerations, where the infrastructure around sample and data handling has become a key factor. In the early 2000s, ease of access for travel was a key parameter. With remote access this is no longer an issue; however, shipping crystals has become a very critical but unpredictable parameter, favouring synchrotrons to which delivery of dewars is reliable. To ensure that crystals are available for a pre-booked shift, we allow two days for shipping. Today, several synchrotrons offer a mail-in service, providing a flexible window for data collection which can compensate for unreliable crystal shipping. Factors such as smooth data transfer and compatibility with our in-house structure repository and LIMS system are also key for enabling an effective workflow from crystal to structure.

**Figure 3:**
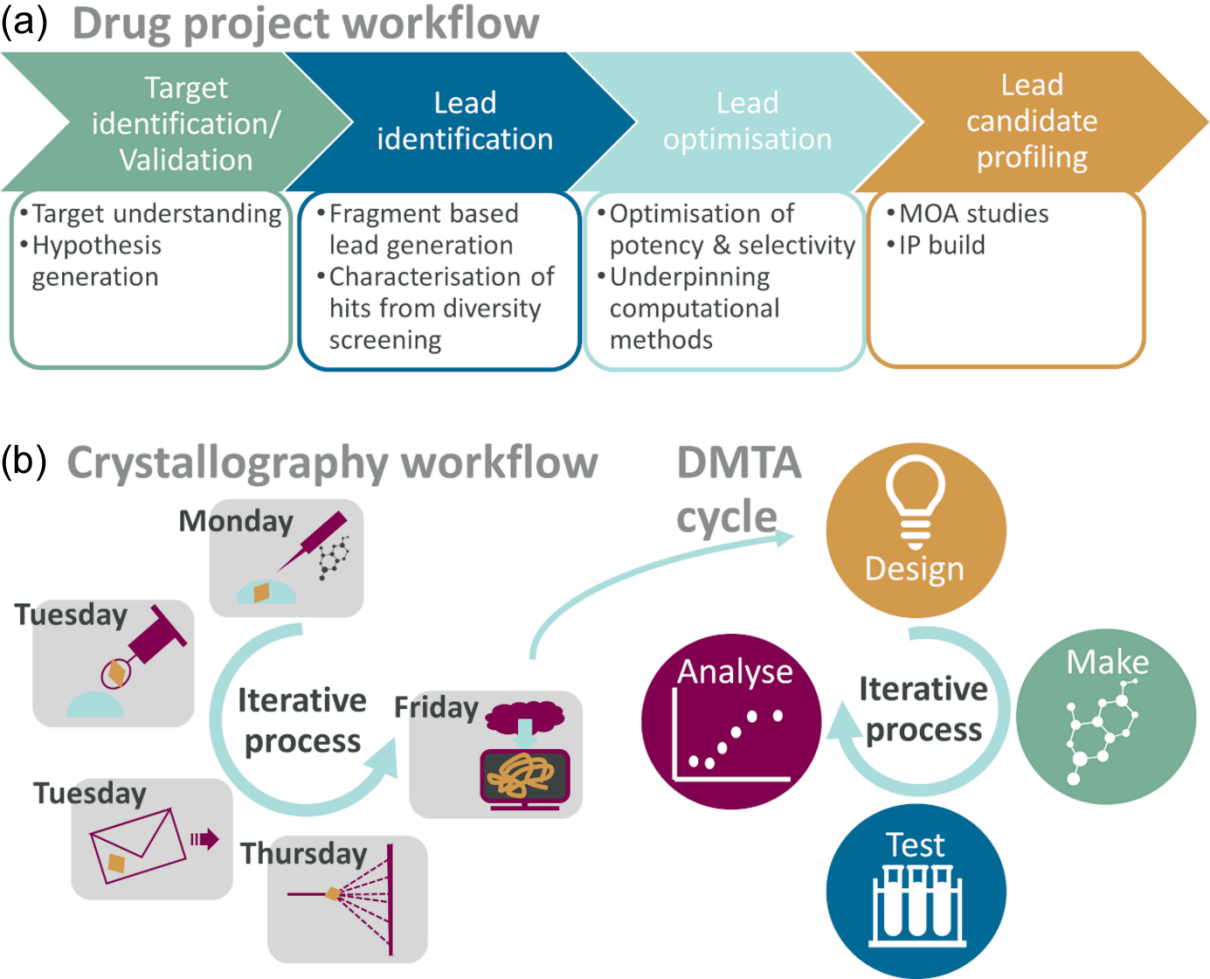
Workflows relevant for structure-based drug design. (*a*) Use of structure during the phases of a drug discovery project. (*b*) Workflow for iterative crystallography linked to the DMTA cycle. The ideal iterative crystallography workflow starts with compound introduction on a Monday followed by crystal harvesting and sample shipment on Tuesday. On Thursday data are collected, and on Friday the data can be retrieved and the structure solved. The structure then feeds into the design phase of the DMTA cycle.

For a robust crystallization protocol yielding crystals that tolerate ligand soaking, we can deliver new structures within a working week from the time we receive the compound. An example is shown in Fig. 3 ▸, where ligand soaking is done on day one, crystal harvesting and shipping on day 2, data collection on day 4, and structure solution and dissemination on day 5. Furthermore, the efficiency of data collection allows 120–160 datasets to be collected in an 8 h shift, with the consequence that capacity for data collection is seldom a bottleneck for structure delivery.

The situation is different when a new crystallization protocol is being developed, or in cases where ligand introduction is challenging. Here direct feedback on the diffraction quality of new crystals, or visualization of the initial map, is desirable. Relying on a ‘synchrotron only’ model, introduces at least three days of delay from crystal harvesting to feedback on experimental outcome. Therefore, such a model is less effective in this situation.

## 3. Impact of structural information across small-molecule drug discovery projects

Access to structural information has the potential to impact a small-molecule drug discovery project throughout the entire discovery phase, from target validation to candidate drug selection, a process that typically takes 4–7 years (Fig. 3 ▸).

When considering a new project for drug discovery, structures of the target protein are used to determine the strategy of the project. For example, a structure-based assessment will underpin the selection of suitable pharmaceutical agents, *e.g.* small-molecule inhibitor, protein degrader or non-small molecule modality (Fauman *et al.*, 2011 ▸; Xie *et al.*, 2023 ▸; Valeur *et al.*, 2017 ▸). Furthermore, structures are routinely used to assess the preferred mode of inhibition or modulation and build hypotheses regarding the biological function. Importantly, structural analysis at this stage of a project relies almost entirely on access to public domain structures and predicted models. The recent advances in structure prediction with *AlphaFold2* (*AF2*) (Evans *et al.*, 2021 ▸) now allow a structure-based hypothesis for every small-molecule project.

The selected target protein is subjected to a selection of methods aimed at finding starting points for drug development. This can be done by rational design using prior knowledge about ligand binding or by employing different screening strategies. The most common approaches include fragment-based lead generation (FBLG) as well as different approaches to diversity screening such as high-throughput screening (HTS), DNA encoded library screening (DEL) and virtual screening (Hughes *et al.*, 2011 ▸; Gironda-Martínez *et al.*, 2021 ▸). While structural information is an integral part of FBLG and a prerequisite for virtual screening, it provides key information when evaluating the outcome from HTS and DEL.

FBLG is a powerful approach that leverages the binding of small, low-molecular-weight compounds (<300 Da) to the target protein. The concept is based on the notion that small fragments, despite having weak affinity for the target, are more likely to make ideal interactions, and combining or elaborating these hits will result in lead-like molecules with optimal interactions (Blundell *et al.*, 2002 ▸). Alongside sensitive biophysical screening methods, such as nuclear magnetic resonance and surface plasmon resonance, macromolecular crystallography plays a crucial role in screening for fragment binders (Davis & Hubbard, 2020 ▸; Schiebel *et al.*, 2016 ▸). This is because soaking crystals at high concentrations of fragments can allow detection of very weak binders, undetectable by other technologies. An added benefit is that the binding mode of the hits will be determined as part of the screening; this information is crucial for developing fragments into more potent lead-like molecules. Fragment libraries for crystallographic screening typically include 100–1000 individual fragments, sometimes combined in cocktails to reduce the number of samples (Cox *et al.*, 2016 ▸; Wollenhaupt *et al.*, 2020 ▸; Lucas *et al.*, 2022 ▸). To be efficient as a screening method and a competitive hit-finding method, fragment screening needs to be executed to deliver chemical lead compounds within a timeframe comparable to that of HTS, for instance. This requires a robust crystallization protocol to be available early in the project. In addition, it is crucial to have an efficient workflow that allows for rapid soaking, harvesting and data collection of hundreds (sometimes thousands) of crystals. To address the latter, fragment screening has been made available as technology platforms at several synchrotron facilities, for both academic groups and industrial research (Lima *et al.*, 2020 ▸; Douangamath *et al.*, 2021 ▸; Wollenhaupt *et al.*, 2021 ▸).

Hit-finding by virtual screening can be run either as large-scale docking or as a similarity search based on known ligands. It offers the opportunity to screen billions of virtual compounds without preparation of physical reagents and is therefore often the first hit-finding approach executed in a project (Bender *et al.*, 2021 ▸; Carlsson & Luttens, 2024 ▸; Zhao, 2024 ▸). Structure-based virtual screening can be particularly useful in cases where there are no good tool compounds available for use in validation experiments. Owing to the timing of this activity, at the very beginning of a project, bespoke experimental in-house structures are seldom available for docking, therefore virtual screens are often based on public domain structures.

Following hit-finding, the hits identified from different screening strategies, for example HTS and DEL, are evaluated for the likelihood of being developed into drug-like molecules with a desirable mode-of-action. A crystal structure of a complex between a hit and its target contributes important information as it allows identification of the binding site. This is particularly valuable if the primary screen is based on binding rather than activity (*e.g.* DEL). Furthermore, the mode-of-action can often be concluded when the structure of the complex is revealed (Li & Kang, 2020 ▸). Importantly, side-by-side analysis of complex structures with hits from all hit-finding methods provide an overview of the chemical space in the context of a given target protein. This makes it possible to assess the potential for compound optimization, *i.e.* if there is scope for specific interactions and if the binding site is compatible with binding of molecules with drug-like properties (Lipinski *et al.*, 2001 ▸; Shultz, 2019 ▸). It is highly desirable to have a well established crystallization protocol in place when the screens are completed to be able to determine the structures within a time frame relevant for selecting which molecules to progress. In addition, an HTS screen may deliver a substantial number of hits/clusters of hits and therefore require capacity allowing for structure determination.

One or a few promising chemical scaffolds from the lead generation activities are selected for optimization with the aim to deliver a clinical candidate. Data from multiple types of assays and predictive models are used to design a compound that is efficacious and safe while having optimal properties for the chosen route of administration and ultimately can be progressed to clinical trials. A framework for tackling this formidable multi-parametric challenge is the so-called design, make, test, analyse (DMTA) cycle, where hypotheses are generated based on the outcome from the previous iteration (Fig. 3 ▸; Plowright *et al.*, 2012 ▸). Structural information provides a cog in this framework by offering inspiration to novel design, validating design hypothesis and underpinning the use of computational drug design methods such as bespoke docking, free-energy perturbation and molecular dynamic simulations (Bassani & Moro, 2023 ▸). To maximize the impact of structural information, structures should ideally be delivered in a timely manner to impact current chemical design. Hence, speed of data generation is important in this phase.

Structural information impacts drug discovery throughout the lifetime of a project. However, each individual structure is not equally influential. For example, the first structure revealing the binding site for a compound class is fundamental for structure-based design. It is not unusual to identify unexpected binding sites (Ludlow *et al.*, 2015 ▸). These sites are hard to reliably identify by computational approaches. In the lead optimization phase, typically small changes are made to a well understood chemical scaffold and structures are solved to answer specific questions. Depending on the nature of the target protein, reliable docking protocols can often be utilized and reduce the need for experimental structures. However, induced fit, conformational changes in the protein upon ligand binding and flipped-binding mode of the inhibitor are examples of consequences of ligand binding that are challenging to predict with computational methods (Olanders *et al.*, 2024 ▸). At the same time these unexpected observations can provide opportunities for novel chemical design (Malhotra & Karanicolas, 2017 ▸).

The requirements for structure delivery thus look different at different stages of a project. For fragment screening it is important to establish a robust system early in the project that allows rapid structure solution for a fragment library typically between 100 and 1000 compounds in size. Similarly, a project using HTS for hit identification may deliver a large batch of compounds for structure studies which requires sufficient capacity at that stage of the project.

In contrast, in lead optimization timing is important, and for effective structure-based design structures should be made available to the project on the timescale relevant to the DMTA cycle. A model allowing frequent and flexible synchrotron access is therefore of priority.

## 4. Real-world data on structure delivery to drug discovery projects

To illustrate delivery of protein structures to small-molecule drug discovery projects, crystallographic data generated at AstraZeneca Gothenburg between 2011 and 2023 were analysed. The total dataset contains 2597 unique structures across 88 projects covering three non-oncology therapy areas.

The projects were classified as ‘high-throughput’, ‘intermediate’ and ‘low-throughput’ based on the number of structures solved during the most productive year (peak year). ‘High-throughput’ is defined as projects with >20 structures delivered in the peak year. ‘Intermediate’ and ‘low-throughput’ projects are defined as projects with 5–20 or <5 structures in the peak year, respectively. 10% of the projects did not yield any structures during the project lifetime and were categorized as ‘none’.

During the period 2011–2023, we supported on average 22 projects each year [Fig. S2(*a*) of the supporting information]. A general but weak trend can be observed towards more ‘high-throughput’ and ‘intermediate’ projects, whereas the number of ‘low-throughput’ projects is declining over time. We attribute this partly to increased frequency and capacity of synchrotron data collection, and the ability to utilize smaller crystals. Other factors influencing the change in distribution between categories over time are improved workflows in protein production and crystallization.

Projects were active in the structure portfolio for up to 9 years, with an average length of 3.2 years [Fig. S2(*b*) of the supporting information]. In total, 6 projects were active for more than 6 years, 4 of these reflect changes in the project objectives during the course of the project, *i.e.* change of preferred mode of action or changed route of administration requiring new chemical equity. Interestingly, we note that projects delivering only a few structures were active for up to 5 years, testifying to the importance of these few structures. Here we find membrane proteins such as PAR2 and LTC4S, for which few, but influential, crystal structures were generated, and MPO where the structure of mechanism-based irreversible inhibitors were studied (Cheng *et al.*, 2017 ▸; Munck af Rosenschöld *et al.*, 2019 ▸; Inghardt *et al.*, 2022 ▸).

**Figure 4:**
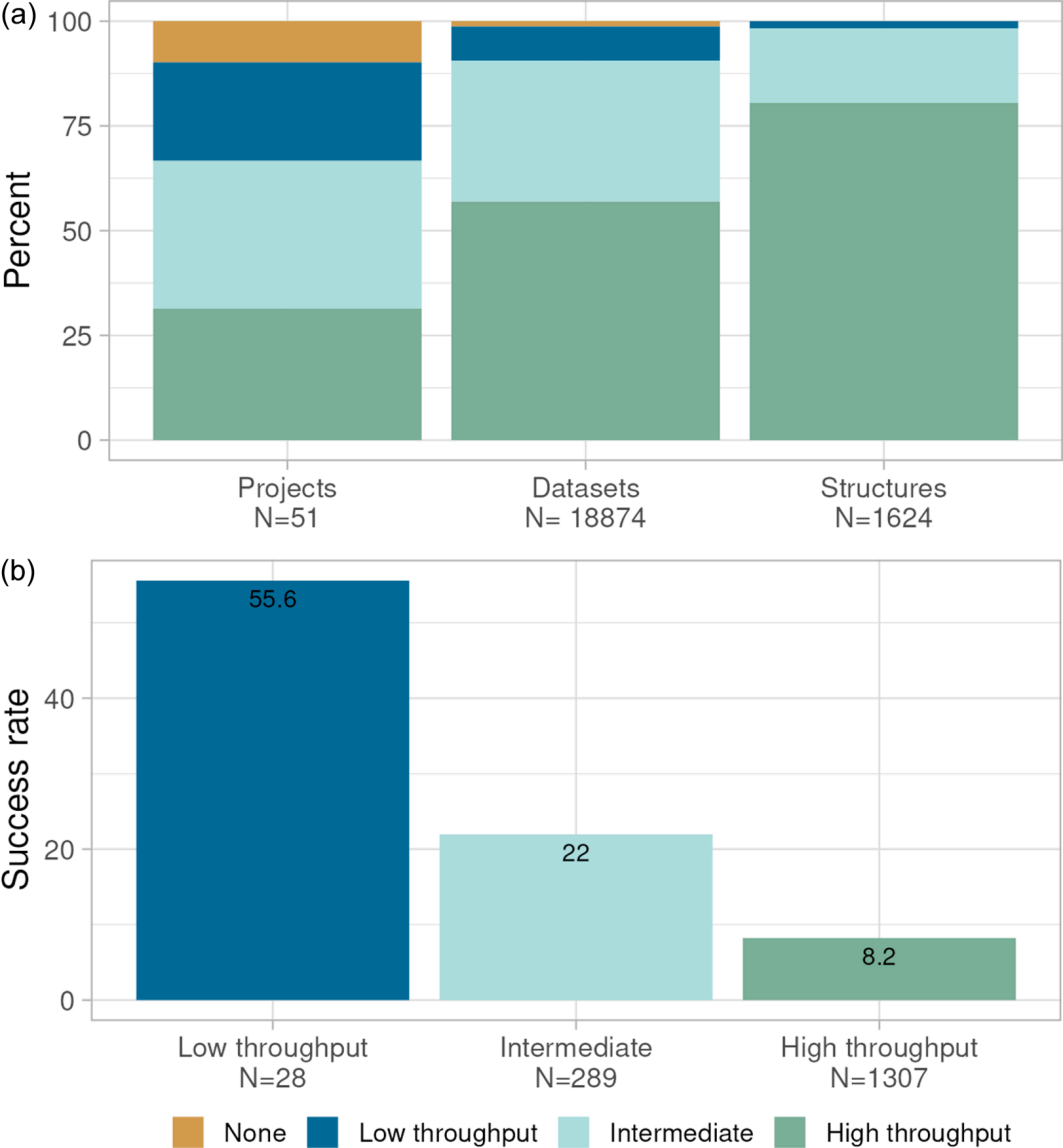
(*a*) Numbers of active projects, datasets and structures recorded in the period 2017–2023. Data are categorized according to project category. (*b*) Overall success rate (number of structures per number of datasets) for different project categories. The ‘high-throughput’ category is defined as projects with >20 structures delivered in the peak year. ‘Intermediate’ and ‘low-throughput’ categories are defined as projects with 5–20 or <5 structures in the peak year, respectively. Projects that did not yield any structures during the project lifetime and were categorized as ‘none’.

To further explore our current working model, relying on synchrotron-only data, we made an analysis of data covering the period 2017–2023. During this period, a total of 18874 datasets were collected resulting in 1624 unique structures supporting 51 projects. Fig. 4 ▸(*a*) shows the data categorized as described previously for projects, number of datasets collected and number of structures solved (as percentages) coloured by category. While the ‘high-throughput’ projects represent only 27% of the total number of projects, they account for 54% of the datasets collected, and almost 80% of the total number of structures. Conversely, the project in the ‘low-throughput’ category accounts for 25% of the total portfolio, but only 9% of the datasets and <2% of the total number of structures. The success rate in terms of number of datasets collected for each solved structure is illustrated in Fig. 4 ▸(*b*). Even for the ‘high-throughput’ projects, 8 datasets are collected per solved structure on average, which may seem high. However, this accounts for testing crystals when the crystallization system is optimized; structures where ligands fail to bind, which often are abundant in the early stages of projects when the ligands typically have low affinity; and non-binders during fragment screening. In addition, it reflects the fact that, even for well established projects, data are collected on more than one crystal to reduce the risk for prolonged delivery times due to variable crystal quality. For the ‘low-throughput’ category, the average number of datasets for each structure is over 55, indicating the effort that goes into this category of projects.

### 4.1. An example of structure delivery across the lifetime of a project

**Figure 5:**
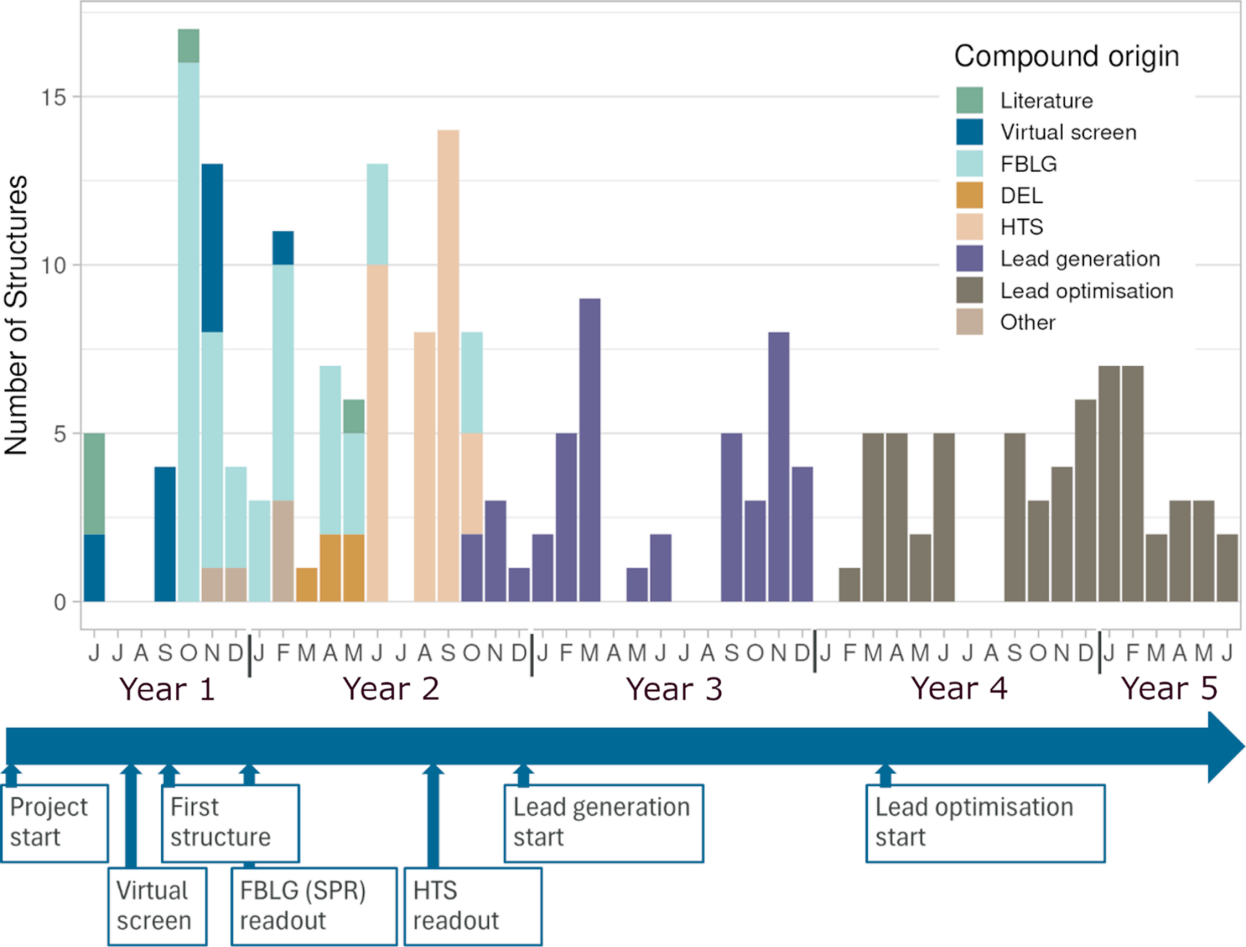
Structure delivery for an example project. The number of structures delivered in the peak year was 72, placing it in the ‘high-throughput’ project category. Structures are coloured according to compound origin. Key project events are indicated on the timeline below the plot.

To exemplify how structures are delivered throughout the project lifetime, we analysed structures generated for a project from our internal pipeline which is currently in a mature state and therefore covers the majority of the discovery phase. The target protein is a soluble enzyme for which the structure had not been described in the literature. Six different protein constructs were evaluated in crystallization, allowing us to establish a robust soaking protocol 6 months after the project started (Fig. 5 ▸). A broad strategy was employed to find chemical starting points for this target. This included ligand-based virtual screening, FBLG, HTS and DEL. Following the structural characterization of a small set of hits from virtual screening, the bulk of the early structures (Q4, Year 1) originates from a fragment screening campaign performed with SPR as the primary screening technology. From the initial SPR screen, 200 fragments displayed a *K*_d_ better than 200 µ*M*. 29 fragments were selected for crystallography, of which 14 delivered a liganded structure. During Year 1, the number of datasets collected per structure is on average 9 (Fig. S4 of the supporting information), reflective of crystal screening during protocol optimization and that most of the compounds during this period are weakly binding fragments. The HTS was completed in Q2 Year 2 and resulted in a large number of hits. 51 compounds representing diverse chemistry were selected for crystallography. Structures could be solved for 35 of these in complex with the target protein. Several HTS compounds displayed binding at unexpected sites, multiple binding sites for the same compound and different types of induced fit, all characteristics that make prediction of binding or docking very difficult. During the later stages of the project, the overall number of structures per month was lower. At this stage the chemical optimization was focused on a few key scaffolds, hence there was a good understanding of how the compounds interact with the target protein and only compounds of specific interest were selected for crystallography. Despite a highly optimized crystal system and more potent compounds, on average more than 4 datasets were collected for each structure, reflecting the variable crystal quality in combination with the desire to deliver structures as soon as possible. In this project structures have been determined for 216 different compounds, out of 270 tested. In total 1230 datasets have been collected and the average resolution for the resulting structures is 2.23 Å.

### 4.2. LTC4 synthase: one project, one structure

**Figure 6:**
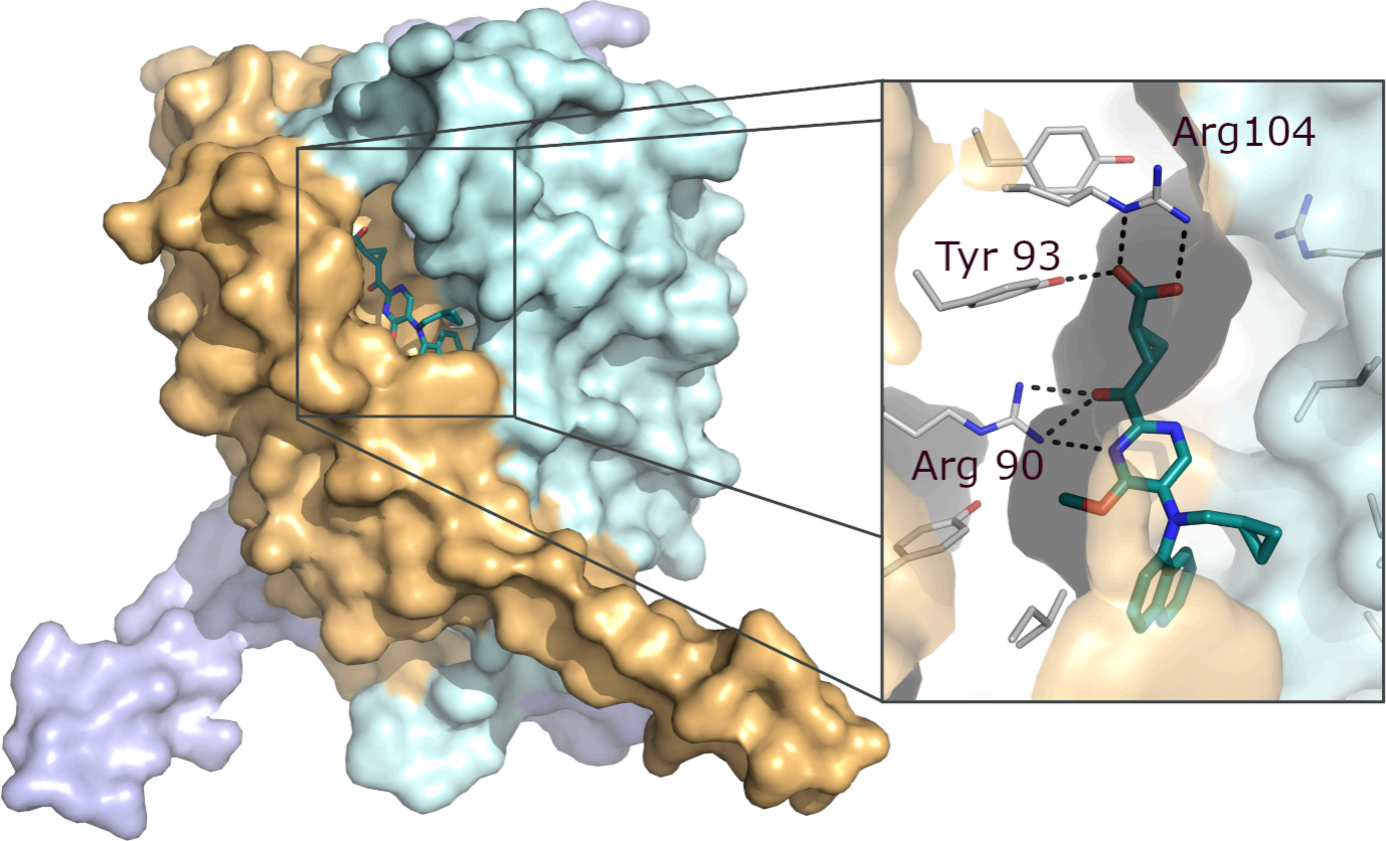
Crystal structure of LTC4S in complex with inhibitor (PDB entry 6r7d).

Leukotriene-C4 synthetase (LTC4S) is a membrane-bound enzyme, central in the cysteine leukotriene cascade, that forms a plethora of proinflammatory and pro-resolution lipid metabolites involved in multiple diseases (Wang *et al.*, 2021 ▸). Following literature precedent (Martinez Molina *et al.*, 2007 ▸), the apo structure could be quickly repeated. However, the crystals were not suitable for soaking, while co-crystallization in the presence of inhibitors under similar conditions was unsuccessful. Optimization of the detergent composition for protein extraction allowed the structure determination of a liganded complex at 2.35 Å resolution (PDB entry 6r7d; Munck af Rosenschöld *et al.*, 2019 ▸). The structure revealed the binding mode of a project compound for the first time (Fig. 6 ▸). Importantly, the structure allowed the chemical design to focus, by guiding the chemical exploration and enabled application of structure-based computational methods that led to the discovery of a candidate drug (AZD9898). Despite considerable effort, no more liganded structures of LTC4S could be generated, but while many projects benefit from iterative crystallography, this project demonstrated the power of a single X-ray structure combined with computational chemistry modelling.

## 5. Discussion

Macromolecular crystallography is an excellent method for structure determination of protein–ligand complexes, as it is fast, highly streamlined and can be repeated for a large number of ligands once a crystallization method is established, and is therefore also cost-effective. Our approach to data collection has evolved in response to the growing availability and capabilities of synchrotron facilities. Starting from infrequent synchrotron data collection requiring travel to the synchrotron facility, combined with in-house data collection, we are now in a situation where 100% of the data is collected at synchrotrons, with weekly remote access. This shift has been driven by the need for timely delivery of structures to support our projects, combined with the ever-improving technology development at synchrotrons. Today, many synchrotrons offer dedicated beamlines suitable for high-throughput crystallography enabling collection of 100s of datasets per day. Important considerations for us as industrial users, beyond the quality of the data and throughput, are ease of access including sample transfer, access to automated data collection workflows, compatibility with in-house databases allowing tracking of the experimental details and short cycle times.

Due to the nature of experimental structures, revealing key interactions between drug-like compounds and their targets, the vast majority of our structures remain key proprietary information. However, at AstraZeneca there is a pronounced strategy to publish results of general interest whenever possible. Between 2004 and 2024, around 110 manuscripts describing 320 structures were published by the AstraZeneca Gothenburg group. However, publications disclosing compounds are often limited to a few protein–ligand structures. Alternatively, in the case of previously unpublished structures, coordinates of the protein alone or in complex with known ligands may be disclosed (Sjögren *et al.*, 2013 ▸; Öster *et al.*, 2024 ▸). The publications thus do not provide an accurate description of how X-ray crystallography brings value to drug discovery.

We have developed workflows to efficiently deliver hundreds of unique X-ray structures per year to a diverse set of drug discovery projects. In order to illustrate how these structures support our portfolio, we have analysed metadata in our structure repository. In the period 2017–2023, where we have relied on synchrotron data only, we have supported 51 projects. Our analysis shows that ‘high-throughput’ projects, defined as the ability to deliver more than 20 structures per year, were achieved in only a relatively low fraction (31%) of our projects. This may be surprising given the recognized importance of high-throughput structural support. Note that key factors such as target validation and considerations for the right patient population are central to target selection, and it is not uncommon that these targets come with limited prospects for achieving a good crystallization system. For example, during the period 2011–2023, 15% of the projects in our structure portfolio lacked reports of structure determination in the literature. Nevertheless, given the recognized impact of even a low number of structures, we initiate structural support for almost all projects with soluble targets. In cases where high-throughput crystallization cannot be achieved, an increased emphasis is placed on solving structures predicted to be key for decision making. The significantly higher number of datasets per successful structure in the ‘intermediate’ and ‘low-throughput’ project categories illustrates the importance of obtaining these key structures.

The role of X-ray crystallography for structure-based drug design has remained largely undisputed throughout the period discussed herein. However, in recent years there has been significant challenge to this notion. In the 2015 commentary Ewen Callaway claimed that ‘The revolution will not be crystallized’, referring to the anticipated impact of cryo-EM on the field of structural biology (Callaway, 2015 ▸). The recent advancements in single-particle cryo-EM have indeed allowed a significant expansion of the target space amenable to structure-based design by enabling structure determination of membrane proteins and large protein complexes (de Oliveira *et al.*, 2021 ▸). However, because of limitations in terms of protein size and throughput, cryo-EM is not a replacement for crystallography, but rather an excellent complement. Thus, many pharmaceutical companies, including AstraZeneca, have made significant investments in the technology. With further technical advancements the balance between crystallography and cryo-EM may shift in the future.

Another development which may change the landscape for X-ray crystallography in structure-based drug discovery are the recent advances in deep-learning-based structure prediction tools, such as *AF2* and *trRosetta*. Structure predictions have reached impressive accuracy, often matching the quality of experimental structures (Jumper *et al.*, 2021 ▸; Yang *et al.*, 2020 ▸). Access to reliable protein or protein-complex models, as generated by *AlphaFold-Multimer*, can significantly impact drug discovery, from underpinning target hypotheses to informing construct design and facilitating molecular replacement structure solutions (Akdel *et al.*, 2022 ▸; Evans *et al.*, 2021 ▸). However, the prediction of binding sites and active conformations has been demonstrated to be less reliable, and care must be taken when using *AF2* models for structure-based design and molecular docking (Terwilliger *et al.*, 2024 ▸; Olanders *et al.*, 2024 ▸). The recently disclosed *AlphaFold3* claims significant improvements in the prediction of protein interactions with small molecules, proteins and nucleic acids (Abramson *et al.*, 2024 ▸), but its impact on real-life drug discovery projects remains to be confirmed, as the software is not currently available for industrial research. Notably, proprietary structure databases, such as our in-house structure repository, provide an extremely valuable source for training of future AI models and thus underpinning improved prediction of the interactions between protein and drug-like molecules.

Ultimately, experimental structures will continue to be important for drug discovery, especially for novel targets with novel mechanisms-of-action or unknown binding sites, but also because of the frequently observed breakdown of the structure–activity relationship due to conformational changes of the target or unexpected binding modes which present challenges for AI-based prediction (Olanders *et al.*, 2024 ▸). Moreover, advances in computational methods, such as prediction of affinity, using free-energy perturbation (Wang, 2024 ▸; Ross *et al.*, 2023 ▸) are making accurate structures an even more important factor for accelerating drug discovery. Coupling the experimental structures with AI-driven generative molecule design solutions, such as REINVENT (Loeffler *et al.*, 2024 ▸), offers another opportunity for faster and more efficient structure-based design.

## 6. Methods

For the metadata analysis, we mined our internal repository for crystal structures. Analysis and plots were done in *R*. Special notes: (1) For the analysis of structure support to small-molecule drug discovery projects, all structures and datasets in support of biologics drug projects, academic projects or publications were eliminated (Fig. 4 ▸, Fig. S2 of the supporting information). (2) Categorization of projects was based on the number of structures solved during the most productive year (peak year) between 2003 and 2024, eliminating the ‘tail’ effect at the start and finish of the period studied, although the number of projects in the higher-throughput categories may be underestimated for the end of the period 2017–2023 (Fig. 4 ▸).

## Supplementary Material

Supporting Figures S1 to S4. DOI: 10.1107/S1600577524012281/he5682sup1.pdf
