# Supplementary material for: Macromolecular crystallography from an industrial perspective – the impact of synchrotron radiation on structure-based drug discovery
Source: J Synchrotron Radiat. 2025 Feb 6;32(Pt 2):294–303. doi: 10.1107/S1600577524012281 (PMC11892899; doi:10.1107/S1600577524012281)
Supplement: Supplementary file 1 [file s-32-00294-sup1.pdf]

JOURNAL OF
SYNCHROTRON
RADIATION

Volume 32 (2025)

Supporting information for article:

Macromolecular crystallography from an Industrial perspective – The impact of synchrotron radiation on structure-based drug discovery

H. Käck and T. Sjögren

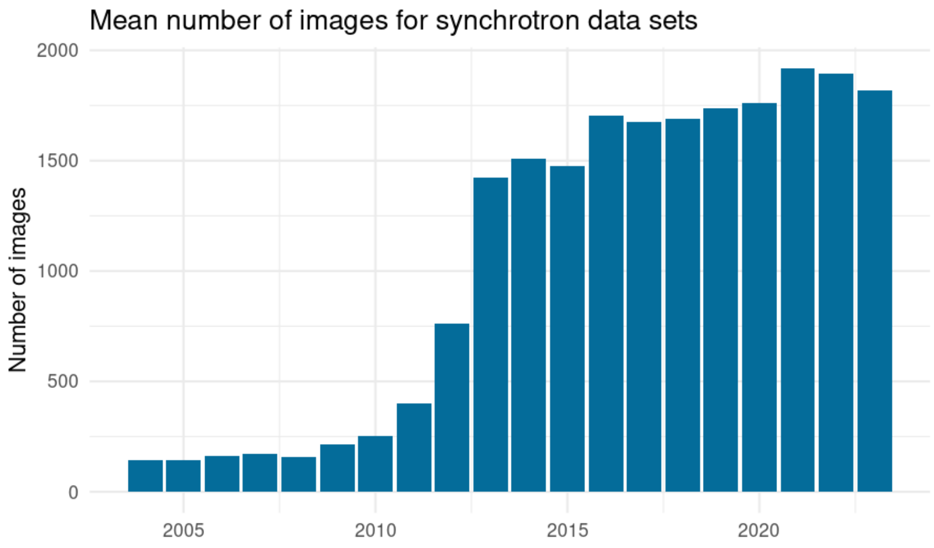

Figure S1 Average number of images per dataset. A dataset was defined as an entry with more than 40 images.

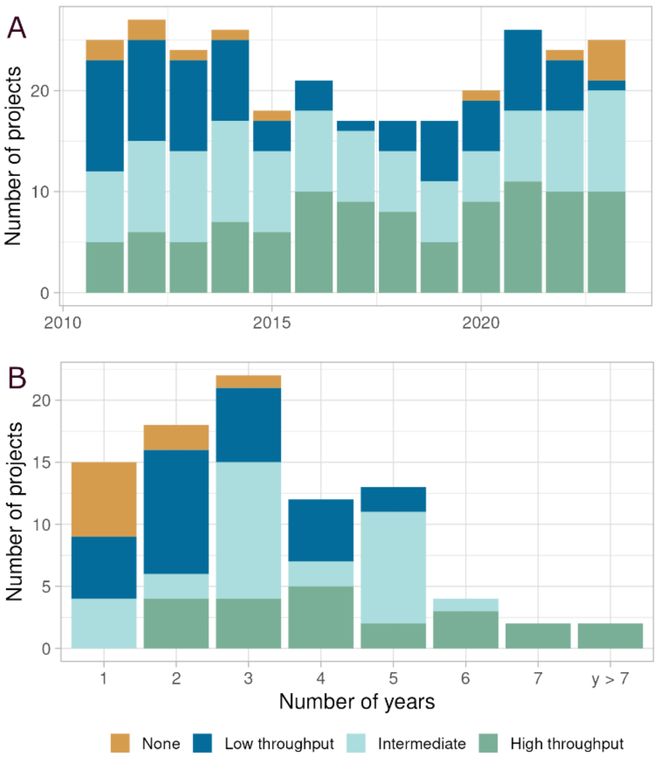

Figure S2 Project support 2011-2023. a) Number of projects per years, coloured by category. b) Number of active years per project coloured by category. The “High throughput” category is defined as projects with >20 structures delivered in the peak year. “Intermediate” and” Low throughput” categories are defined as projects with 5-20 or less than 5 structures in the peak year respectively. Projects which did not yield any structures during the project lifetime and were categorised as “None”.

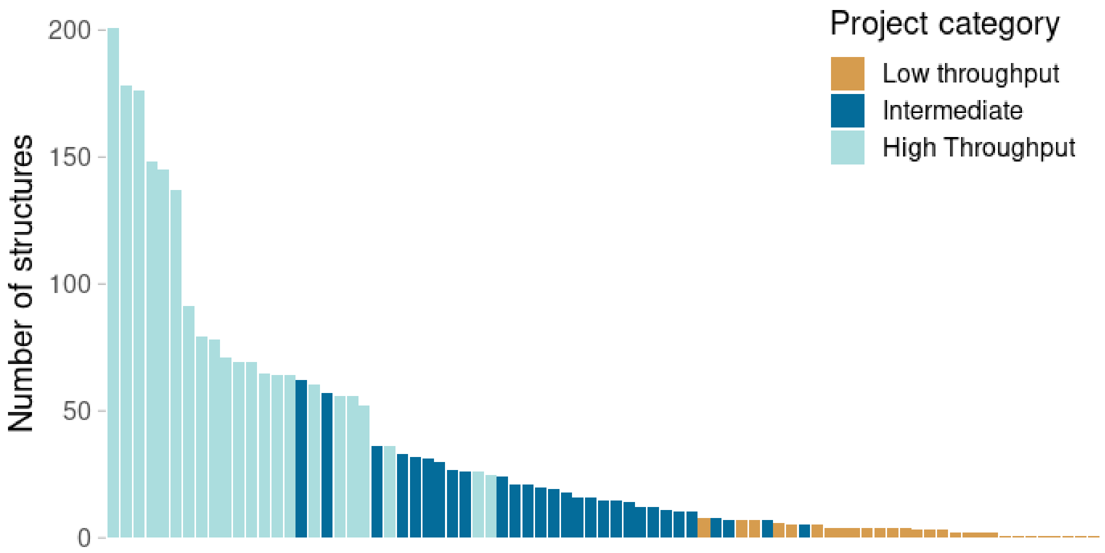

Figure S3 Total number of structures delivered per project 2011-2023. Projects are colored according to category. The "High throughput" category is defined as projects with >20 structures delivered in the peak year. "Intermediate" and" Low throughput" categories are defined as projects with 5-20 or less than 5 structures in the peak year respectively. Projects which did not yield any structures during the project lifetime and were categorised as "None".

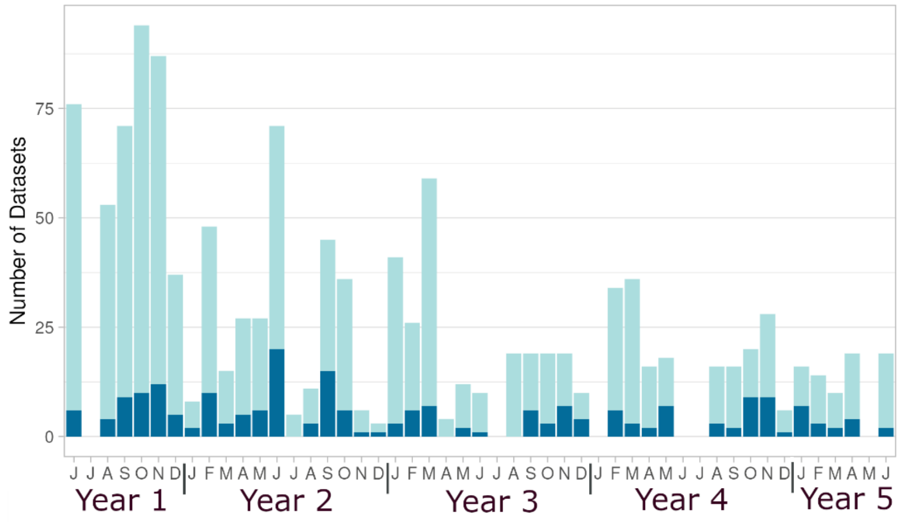

Figure S4 Data sets collected per month for an example project. Datasets resulting in a unique structure are shown in dark blue.
